# NAT10 promotes proliferation, invasion, and epithelial–mesenchymal transition in hepatocellular carcinoma through α-tubulin acetylation

**DOI:** 10.1080/19336918.2026.2697609

**Published:** 2026-08-24

**Authors:** Haogang Yu, Kankai Zhu, Guoliang Zhang, Yang Dong, Xiaodong Wang, Shufen Zhang, Jiajing Huang, Xuemei Lu, Xiuming Zhang, Hao Liu, Wei Chen

**Affiliations:** a Department of Radiotherapy, The First Affiliated Hospital, School of Medicine, Zhejiang University, Hangzhou, Zhejiang, China; b Department of Gastrointestinal Surgery, The First Affiliated Hospital, School of Medicine, Zhejiang University, Hangzhou, Zhejiang, China; c Department of Anorectal Surgery, The First Affiliated Hospital, School of Medicine, Zhejiang University, Hangzhou, Zhejiang, China; d Cancer Institute of Integrated Traditional Chinese and Western Medicine, Key Laboratory of Cancer Prevention and Therapy Combining Traditional Chinese and Western Medicine of Zhejiang Province, Zhejiang Academy of Traditional Chinese Medicine, Tongde Hospital of Zhejiang Province, Hangzhou, Zhejiang, China; e Department of Pathology, The First Affiliated Hospital, College of Medicine, Zhejiang University, Hangzhou, Zhejiang, China; f Provincial Key Laboratory of Precise Diagnosis and Treatment of Abdominal Infection, Sir Run Run Shaw Hospital, School of Medicine, Zhejiang University, Hangzhou, Zhejiang, China

**Keywords:** Hepatocellular carcinoma, NAT10, acetylation, α-tubulin

## Abstract

Hepatocellular carcinoma (HCC) remains a global health challenge due to its high metastatic potential and poor prognosis. This study investigated the role and underlying molecular mechanisms of *N*-acetyltransferase 10 (NAT10)-mediated α-tubulin acetylation in HCC progression. Using Huh-7 and SNU-449 cell lines, we established stable knockdown and overexpression models to systematically evaluate both gain- and loss-of-function effects. Functional assays revealed that NAT10 suppression significantly decreased α-tubulin acetylation, thereby inhibiting cell proliferation, invasion, and migration. Conversely, NAT10 overexpression promoted these oncogenic traits. In vivo validation confirmed NAT10 accelerates tumor growth and lung metastasis. Treatment with the NAT10 inhibitor Remodelin partially reversed the phenotypic effects of NAT10 overexpression and restored epithelial – mesenchymal transition (EMT) marker expression. In conclusion, our results demonstrate that NAT10 drives HCC progression by regulating the α-tubulin acetylation axis, highlighting its potential as a promising therapeutic target for combating HCC metastasis.

## Introduction

Hepatocellular carcinoma (HCC), with a high mortality rate, is the most prevalent malignant tumor of the liver. Recent times have reported a steadily increasing incidence in HCC worldwide. HCC was reportedly the sixth most common cancer type and the third leading cause of cancer-related deaths in 2022 [1]. Various factors are responsible for the development of liver cancer, such as chronic infection by hepatitis B virus, liver cirrhosis, alcohol abuse, and metabolic syndrome [2]. These factors cause proliferation and apoptosis of liver cells through distinct biological mechanisms, resulting in liver cancer. Although surgical resection, targeted therapy, and liver transplantation can prolong patient survival, the prognosis for HCC remains relatively poor because of the high rates of tumor recurrence and metastasis [3,4]. Therefore, it is essential to identify effective intervention targets and conduct further investigation into the regulatory mechanisms responsible for HCC recurrence and metastasis in order to improve the prognosis of patients with HCC.

*N*-acetyltransferase 10 (NAT10) is a nucleolar protein comprising 872 amino acids and possessing histone acetylation activity. It contains an acetyltransferase domain and a lysine-rich carboxy-terminal region [5]. NAT10 has been implicated in several critical cellular processes, including DNA damage repair, ribosomal RNA (rRNA) transcription activation, cell division, and microtubule acetylation [6]. Research indicates that NAT10 undergoes intracellular redistribution and is overexpressed in tumor tissues, such as those involved in melanoma, liver cancer, nasopharyngeal carcinoma, and gastric cancer, closely correlating with tumor prognosis [7–10]. Zhang et al. demonstrated that inhibition of the activity of GSK-3β, a protein kinase, in colorectal cancer promotes the translocation of NAT10 from the nucleus to the cell membrane, thereby activating the Wnt/β-catenin signaling pathway, which enhances tumor cell activity through two pathways: promoting epithelial – mesenchymal transition (EMT) and cytoskeletal remodeling. This enhanced tumor cell activity facilitates the invasion and metastasis of colorectal cancer [11]. Additionally, Song et al. found that the downregulation of NAT10 reduces the levels of *N*4-acetylcytidine in gastric cancer and inhibits AKT phosphorylation and EMT [12]. Our previous studies have also demonstrated that NAT10 promotes HCC resistance to doxorubicin by regulating the EMT [13]. EMT is an important biological behavioral change that occurs during tumor invasion and metastasis and it suggests a potential association between NAT10 and the malignant progression and metastasis in HCC. However, the specific mechanisms underlying NAT10-mediated invasion and metastasis in HCC remain poorly defined.

Acetylation is a reversible post-translational modification process that primarily involves the transfer of acetyl groups to lysine residues via acetyltransferases. NAT10, an important acetyltransferase *in vivo*, exhibits histone acetylase activity and plays a role in regulating the occurrence and progression of various tumors through epigenetic modifications. Beyond its effects on histones, acetylation also influences the activity of non-histone proteins [14]. Furthermore, accumulating evidence has demonstrated a direct association between NAT10 and α-tubulin acetylation. Shen et al. reported that NAT10 localizes to the midbody and regulates cytokinesis by acetylating microtubules, identifying α-tubulin as a principal substrate [15]. Tan et al. further showed that the loss of nucleolar localization of NAT10, accompanied by its redistribution to the cytoplasm and plasma membrane, enhances HCC cell migration and invasion through increased α-tubulin acetylation [16]. Although these studies established a link between NAT10-mediated α-tubulin acetylation and the metastatic potential of HCC, the causal contribution of this signaling axis to EMT, tumor growth, and distant metastasis, as well as its potential as a therapeutic target, remains insufficiently defined. Therefore, based on the established connection between NAT10 and α-tubulin acetylation in HCC, we hypothesize that targeting this regulatory axis may represent a novel strategy to suppress disease progression.

Microtubules are protein polymers formed by the assembly of microtubule proteins, primarily consisting of α-tubulin and β-tubulin. As a key component of microtubules, α-tubulin is significantly associated with tumor prognosis. Existing studies indicate that tumor development, uncontrolled cell proliferation, and the malignant behaviors of tumor cells, such as EMT, invasion, and metastasis, are closely linked to the abnormal expression and distribution of α-tubulin. Abnormal α-tubulin expression has been observed in tumor tissues involved in various cancers, such as gastric, rectal, prostate, and lung cancers [17–19]. Acetylation of lysine 40 on α-tubulin stabilizes microtubules within cells. Acetylated α-tubulin (ACL-α-tubulin) can modulate the movement and binding of motor protein-1, affecting the motility and adhesion of tumor cells, thereby promoting tumor invasion and metastasis [20]. Barcellos et al. demonstrated that ARHGAP21 can upregulate the acetylation level of α-tubulin, facilitating EMT in various tumor cells and enhancing their invasive capabilities [21].

This study aims to investigate the role of NAT10 in promoting α-tubulin acetylation and examine its effects on the invasive and migratory behaviors of HCC cells. Employing a combination of cell culture techniques, RNA interference, Western blotting, and *in vivo* models, we systematically analyze the functional consequences of NAT10 expression on α-tubulin acetylation in HCC. This research not only seeks to elucidate the intricate molecular mechanisms underlying HCC invasion and metastasis, but also aims to provide a foundation for developing targeted therapies that could improve the prognosis for patients afflicted by this aggressive malignancy.

## Methods

### Cell culture

Huh-7 and SNU-449 were purchased from the American Type Culture Collection (ATCC). All cell lines were confirmed to be free from mycoplasma contamination. Huh-7 was incubated in Dulbecco’s modified Eagle’s medium (DMEM) (Gibco, Grand Island, NY, USA), supplemented with 10% fetal bovine serum (FBS) (Gibco) at 37°C with 5% CO_2_. The SNU-449 cell line was seeded in the 1640 medium containing 10% FBS supplemented with 100 U/mL of penicillin/streptomycin. Remodelin, an inhibitor of NAT10, was purchased from Selleck (Shanghai, China). The HCC cells were pretreated with Remodelin (1 μM) for 24 h.

### Reverse transcription quantitative polymerase chain reaction (RT-qPCR)

TRIzol® reagent (Invitrogen, Carlsbad, CA, USA) was employed for extracting total RNA from the HCC cells. The extracted RNA was then reverse-transcribed into complementary DNA (cDNA) using a reverse transcription kit from Takara (Japan). For qRT-PCR analysis, SYBR Premix Ex TaqII (Takara, Japan) was used according to the manufacturer’s protocols. PCR primers specific to NAT10 and GAPDH were utilized for the amplification process. The following primer sequences were employed: NAT10: F: 5′-ATAGCAGCCACAAACATTCGC; R: 5′-ACACACATGCCGAAGGTATTG; GAPDH: F: 5′-GCGGGGCTCTCCAGAACATC; R: TCCACCACTGACACGTTGGC.

### Cell transfection

siRNA targeting NAT10 (si-NAT10; si-NAT10#1: sense GCUAGUGGUCAUCCUCCUATT, antisense UAGGAGGAUGACCACUAGCTT; si-NAT10#2: sense CCAUCUCUCGCAUCUAUUUTT, antisense AAAUAGAUGCGAGAGAUGGTT; si-NAT10#3: sense GCAUGGACCUCUCUGAAUATT, antisense UAUUCAGAGAGGUCCAUGCTT) and negative control siRNA (si-NC) (GenePharma Co., Ltd., Shanghai, China) were employed. A NAT10-overexpressing lentiviral construct and the corresponding empty vector control were obtained from HANBIO Biotechnology Co., Ltd. (Shanghai, China). Huh-7 and SNU-449 cells were seeded in six-well plates and cultured to 70–80% confluence before transfection. Cells were transfected with si-NAT10, si-NC, NAT10 lentivirus, or empty vector using Lipofectamine 3000 Transfection Reagent (Thermo Fisher Scientific, Waltham, MA, USA) according to the manufacturer’s instructions. After 48 h, cells were harvested for subsequent analyses. The efficiency of NAT10 knockdown and overexpression was confirmed by RT-qPCR and Western blotting.

### Western blot analysis

HCC cells were lysed on ice for 30 min in RIPA lysis buffer (Beyotime Biotechnology, Shanghai, China) supplemented with protease inhibitors according to the manufacturer’s instructions. Protein concentrations were determined using a bicinchoninic acid assay kit (Beyotime). Equal amounts of protein were separated by 10% sodium dodecyl sulfate – polyacrylamide gel electrophoresis and transferred onto polyvinylidene fluoride membranes (Millipore, Billerica, MA, USA). After blocking with 5% nonfat milk in Tris-buffered saline containing 0.1% Tween-20 for 2 h at room temperature, membranes were incubated overnight at 4°C with the following primary antibodies: anti-NAT10 (Cat. No. 13,365–1-AP; Proteintech, Wuhan, China), anti-α-tubulin (Proteintech, Wuhan, China), anti-acetyl-α-tubulin (Thermo Fisher Scientific), anti-E-cadherin (Cat. No. 3195S; Cell Signaling Technology, Danvers, MA, USA), anti-Vimentin (Cat. No. 5741S; Cell Signaling Technology), anti-ZEB1 (Cat. No. 70512S; Cell Signaling Technology), anti-TWIST (Cat. No. 90445S; Cell Signaling Technology), anti-Snail (Cat. No. 3879S; Cell Signaling Technology), and anti-GAPDH (Cell Signaling Technology). Following three washes with Tris-buffered saline containing 0.1% Tween-20, membranes were incubated with the appropriate horseradish peroxidase – conjugated secondary antibodies for 1 h at room temperature. Protein bands were visualized using an enhanced chemiluminescence detection kit (Millipore) and quantified by densitometric analysis.

### Immunofluorescence (IF) analysis

Acetyl-α-tubulin, E-cadherin, and Vimentin were detected in different groups of the transfected HCC cells. The cells were cultured in 24-well plates with slides and fixed with 4% paraformaldehyde for 30 min. They were then permeated with 0.1% Triton X-100 for 20 min and incubated overnight with primary antibodies against E-cadherin and Vimentin at 4°C, followed by incubation with secondary antibodies at room temperature for 1.5 h. The nucleus was stained with DAPI and imitated with confocal microscopy (LSM 880; Carl Zeiss AG, Oberkochen, Germany).

### 5-ethynyl-2′-deoxyuridine (EdU) assay

EdU assay was performed using an EdU kit (Invitrogen, Carlsbad, CA, USA). HCC cells (5 × 10^3^) were inoculated into 96-well plates one day in advance. They were then fixed with 4% paraformaldehyde and treated with 0.3% Triton X-100. After co-incubation with the Click reaction solution according to the manufacturer’s instructions, the cells were treated with the Hoechst solution for 30 min. Images were obtained using a fluorescence microscope (Olympus, Tokyo, Japan).

### Transwell assay

The upper chamber of a Transwell plate was pre-coated with a diluted matrix gel (60 μL, BD Biosciences, San Jose, CA, USA) at 37°C for 30 min. The cells (1 × 10^5^, FBS-free medium (200 µL) were added to each upper chamber of the Transwell plate (Corning Costar, Corning, NY, USA). Next, 700 µL of the medium containing 10% FBS was filled into each lower chamber of the Transwell plate. After incubation for 24 h, the cells were fixed and stained with 4% paraformaldehyde and crystal violet solutions. The upper room was cleaned with a cotton ball. The migrated cells on the lower surface were photographed with a microscope and counted in five randomly selected fields of view of a 200× microscope.

### Wound healing assay

The migration ability of different groups of HCC cells was examined through a wound-healing assay. Briefly, the cells were seeded at a concentration of 4 × 10^5^ cells/well in six-well plates. After reaching 100% confluence, a scratch was created on the cell monolayer with a 10-µL pipette tip. The movement of different groups of HCC cells was measured at 0 and 48 h under a microscope (Olympus, Tokyo, Japan).

### In vivo models

All animal experiments were approved by the Ethical Committee of the First Affiliated Hospital of Zhejiang University, Hangzhou, China. Male BALB/c nude mice (4–6-week old) were used to establish subcutaneous xenograft models. Next, 24 mice were randomly divided into four groups. The Huh-7 cells stably transduced with lentivirus carrying shNAT10, shNC, oeNAT10, or vector were suspended in PBS at a density of 1 × 10^6^ cells/100 μL and subcutaneously injected into the right axilla region of the mice. The body weight and tumor dimensions (length [L] and width [W]) were measured every two days. Tumor volume (mm^3^) was calculated using the formula: Volume = (L × W^2^)/2. When the tumor volume of any group reached 100–1500 mm^3^, all mice were euthanized, and xenograft tumors were excised for further analysis.

For detecting metastasis, the Huh-7 cells were transfected with shNAT10, shNC, oeNAT10, or vector, and subsequently labeled with luciferase to enable *in vivo* tracking. Following the labeling, these modified cells were injected into the tail vein of nude mice; six mice were assigned to each experimental group. Progression of tumors and lung metastases was monitored using the Lumina III Imaging System (PerkinElmer, Waltham, Massachusetts, USA).

### Hematoxylin and eosin (H&E) staining

First, tumor tissues from mice were rapidly fixed in 10% formalin. The tissues were then dehydrated and embedded in paraffin blocks. Next, 4-μm-thick sections were prepared, dewaxed, and rehydrated. The nuclei were stained with hematoxylin (5–10 min), differentiated in hydrochloric acid – alcohol solution (5–10 s), and blued in ammonia water (30 s to 1 min). The cytoplasm was counterstained with eosin (1–2 min). Finally, the sections were dehydrated, cleared, mounted, and observed under a microscope.

### Immunohistochemistry

Following the instructions of the Ki67 Cell Proliferation Detection Kit (Beyotime), 4-μm-thick tissue sections were dewaxed and rehydrated, followed by incubation with a fixation solution for 5–15 min. Next, an immunostaining blocking buffer was applied and incubated at room temperature for 10–20 min. Subsequently, mouse monoclonal anti-Ki67 antibodies were added and incubated overnight at 4°C. After washing with PBS, anti-mouse Cy3-conjugated secondary antibodies were incubated at room temperature for 1 h. The nuclei were stained with DAPI solution for approximately 5 min at room temperature. The sections were mounted with coverslips and observed under a fluorescence microscope.

### Statistical analysis

All the statistical analyses were conducted by using GraphPad Prism 8.0 (GraphPad, USA). The two groups were compared using the unpaired student’s *t*-test. The data were presented as mean ± standard deviation. *p* < .05 was considered statistically significant.

## Results

### Expression and prognostic significance of NAT10 in HCC

**Figure 1.**
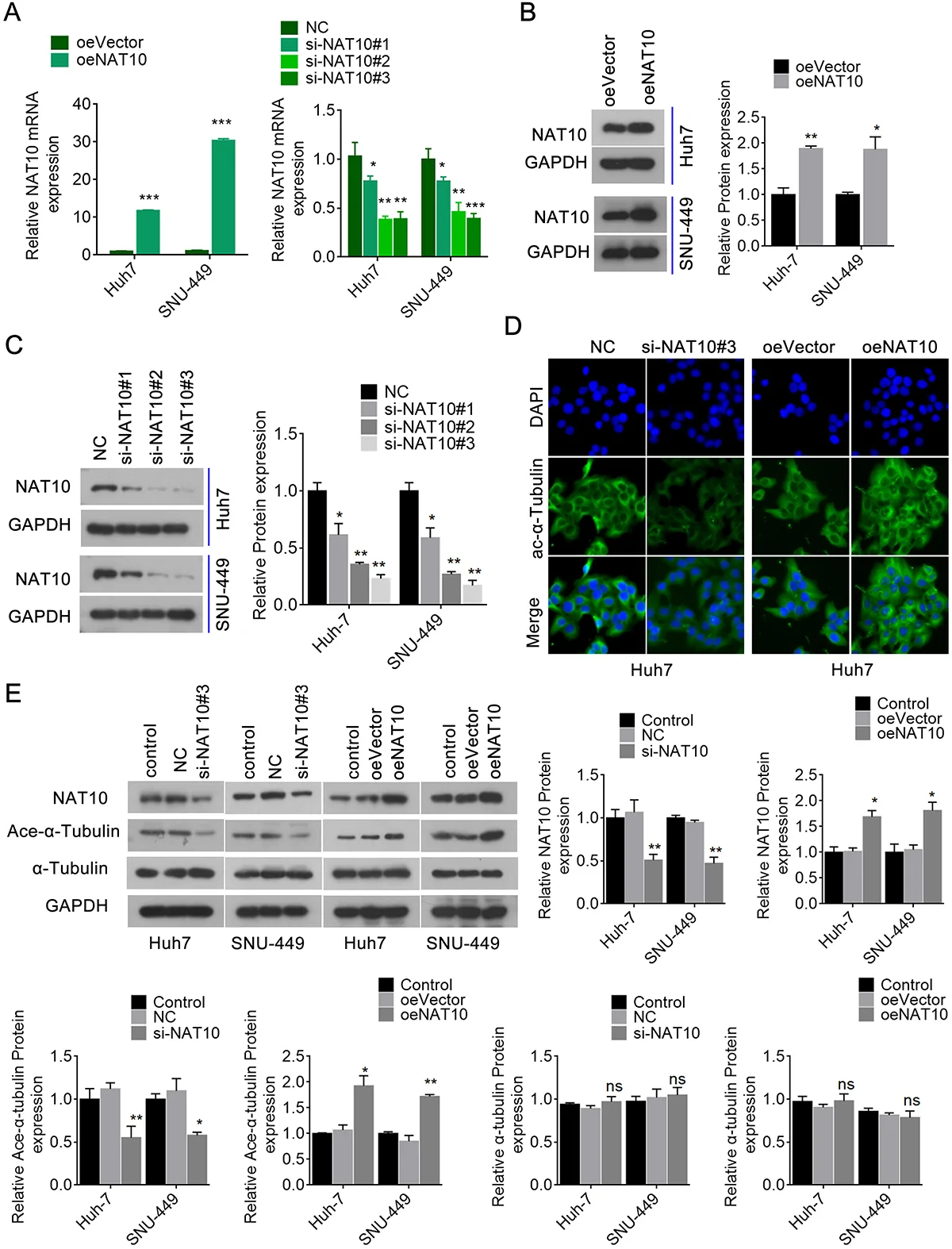
Effect of NAT10 on α-tubulin acetylation in HCC cells. (A) RT-qPCR analysis showing NAT10 expression in Huh7 and SNU-449 cells transfected with a NAT10 overexpression plasmid or NAT10-targeting siRnas (si-NAT10-1, si-NAT10-2, and si-NAT10-3). (B – C) Western blot analysis confirming NAT10 protein levels in Huh7 and SNU-449 cells following transfection with the NAT10 plasmid or NAT10 siRnas. (D) Immunofluorescence staining illustrating changes in acetylated α-tubulin fluorescence intensity in the si-NAT10-3 and NAT10-overexpression groups compared with their respective controls. (E) Western blot analysis demonstrating the relationship between NAT10 expression and acetylated α-tubulin levels.

To assess variations in NAT10 expression between HCC tissues and their adjacent counterparts, we examined NAT10 expression levels in 371 patients with HCC and 50 healthy individuals by utilizing the UALCAN database (http://ualcan.path.uab.edu/). As depicted in Figure S1A, the expression of NAT10 in patients with HCC was markedly elevated compared to that in normal healthy subjects. For a better understanding of the influence of NAT10 on the prognostic outcomes of patients with HCC, we leveraged the data from the TCGA LIHC database to investigate the correlation between NAT10 expression and the clinical prognosis of patients with HCC through a Kaplan – Meier survival analysis. As illustrated in Figure S1B, the overall survival (OS) rates for patients exhibiting a high NAT10 expression were significantly lower than those in patients with low NAT10 levels. These results underscore the prognostic significance of NAT10 in HCC, as well as its potential oncogenic implications. Subsequently, we employed Western blotting and RT-PCR to assess the expression levels of NAT10 in six HCC lines: SNU-449, Huh-7, SNU-387, Hep3B, SNU-475, and sk-hep-1. The findings revealed that SNU-449 had the highest NAT10 expression, while Huh-7 had the lowest (Figures 1(C-E)). Therefore, we chose SNU-449 and Huh-7 to perform the subsequent experiments.

NAT10,*N*-acetyltransferase 10; RT-qPCR, reverse transcription quantitative polymerase chain reaction.

### Effect of NAT10 on α-tubulin acetylation in HCC cells

NAT10 functions as an acetyltransferase, and previous studies have demonstrated its role in maintaining microtubule stability through α-tubulin acetylation, thereby promoting cell proliferation [15]. To determine whether NAT10 regulates tubulin acetylation in HCC, Huh-7 and SNU-449 cells were transfected with a NAT10 overexpression plasmid or NAT10-targeting siRNAs (si-NAT10-1, si-NAT10-2, and si-NAT10-3). RT-qPCR and Western blot analyses confirmed that NAT10 expression was markedly reduced following siRNA-mediated silencing and significantly increased after plasmid transfection in both cell lines (Figure 1(A–C)). Among the three siRNAs tested, si-NAT10-3 exhibited the highest knockdown efficiency and was therefore used in subsequent experiments. We next examined α-tubulin acetylation levels by immunofluorescence and Western blotting. Immunofluorescence analysis demonstrated a clear reduction in acetylated α-tubulin fluorescence intensity in the si-NAT10-3 group, whereas NAT10 overexpression led to a pronounced increase compared with their respective controls (Figure 1(D)). Consistently, Western blot results showed that NAT10 silencing decreased acetylated α-tubulin levels, while NAT10 overexpression enhanced its acetylation (Figure 1(E)). To further assess the clinical relevance of these findings, we analyzed nine paired HCC tissues and adjacent normal tissues. Western blot analysis revealed significantly elevated NAT10 protein expression in tumor tissues, which positively correlated with increased α-tubulin acetylation (Figure S2). Collectively, these data indicate that NAT10 regulates α-tubulin acetylation in HCC and may contribute to disease progression through modulation of microtubule dynamics.

### NAT10 promotes proliferation, invasion, and migration of HCC cells

**Figure 2.**
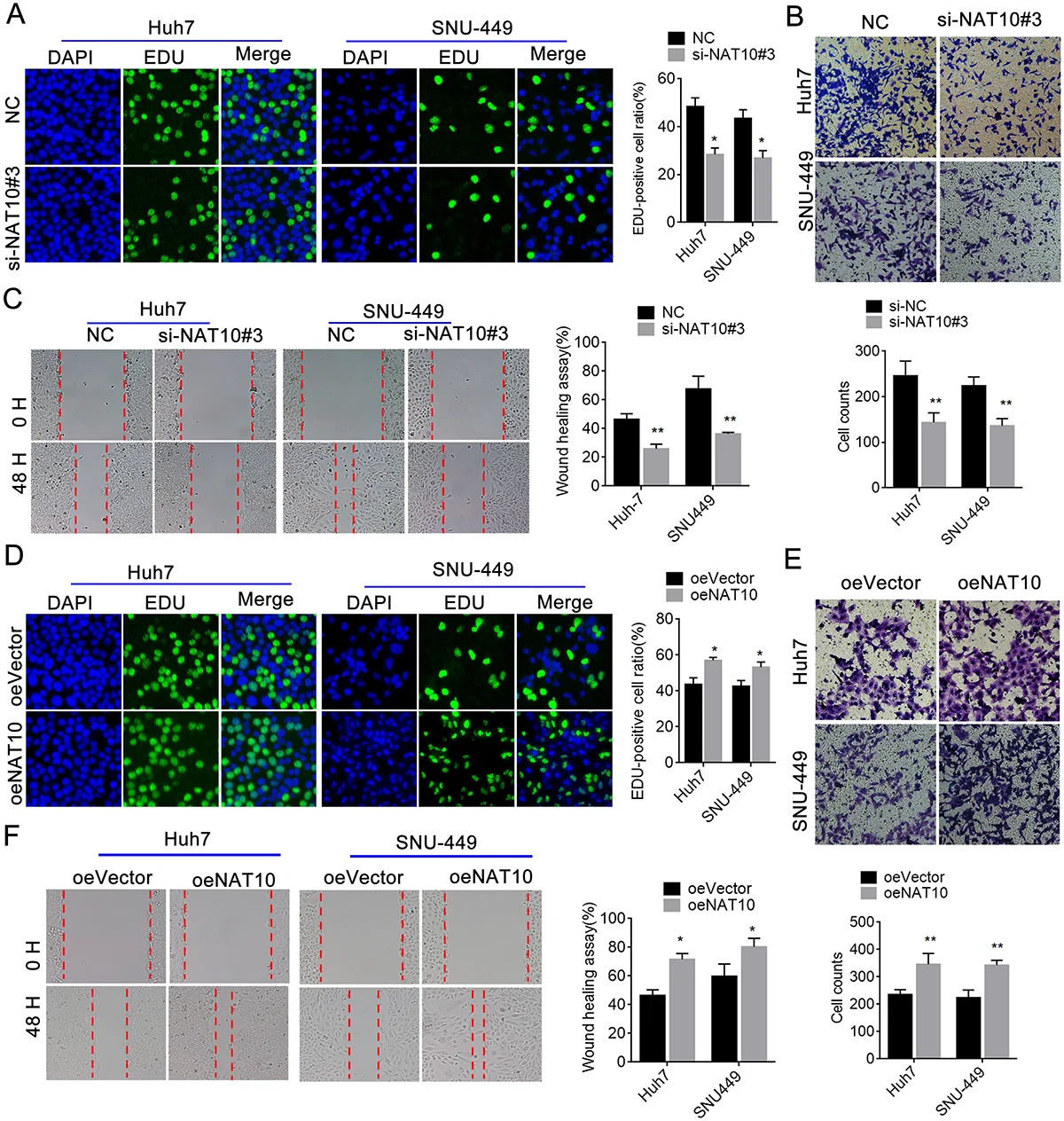
Role of NAT10 in proliferation, invasion, and migration of HCC cells. (A) EdU assay assessing cell proliferation in HCC cells with reduced NAT10 expression compared to that in the NC group. (B Transwell and (C) Wound healing assays evaluating invasion and migration of HCC cells treated with NAT10 siRNA versus NC. (D – F) Analysis of proliferation, invasion, and migration of Huh7 and SNU-449 cells transfected with the NAT10 plasmid compared to those of controls.

EdU, 5-ethynyl-2′-deoxyuridine; HCC, hepatocellular carcinoma; NAT10,*N*-acetyltransferase 10; NC, negative control.

Next, we explored the biological role of NAT10 in HCC by assessing its effects on the proliferation, invasion, and migration of the cancer cells. The EdU assay demonstrated that reduced NAT10 expression significantly decreased the cell proliferation compared to that in the NC group (Figure 2(A)). Additionally, transwell and wound healing assays showed that fewer NAT10 siRNA-treated HCC cells traversed the membrane and had a larger wound distance compared to that in the NC group (Figures 2(B,C)). Conversely, transfection of Huh7 and SNU-449 cells with the NAT10 plasmid resulted in an increased NAT10 expression, which promoted enhanced proliferation, invasion, and migration of the cancer cells compared to those observed in the NC group (Figures 2(D–F)). These findings suggest that NAT10 functions as an oncogene in the proliferation, invasion, and migration of HCC cells.

### Effect of NAT10 on EMT in HCC cells

**Figure 3.**
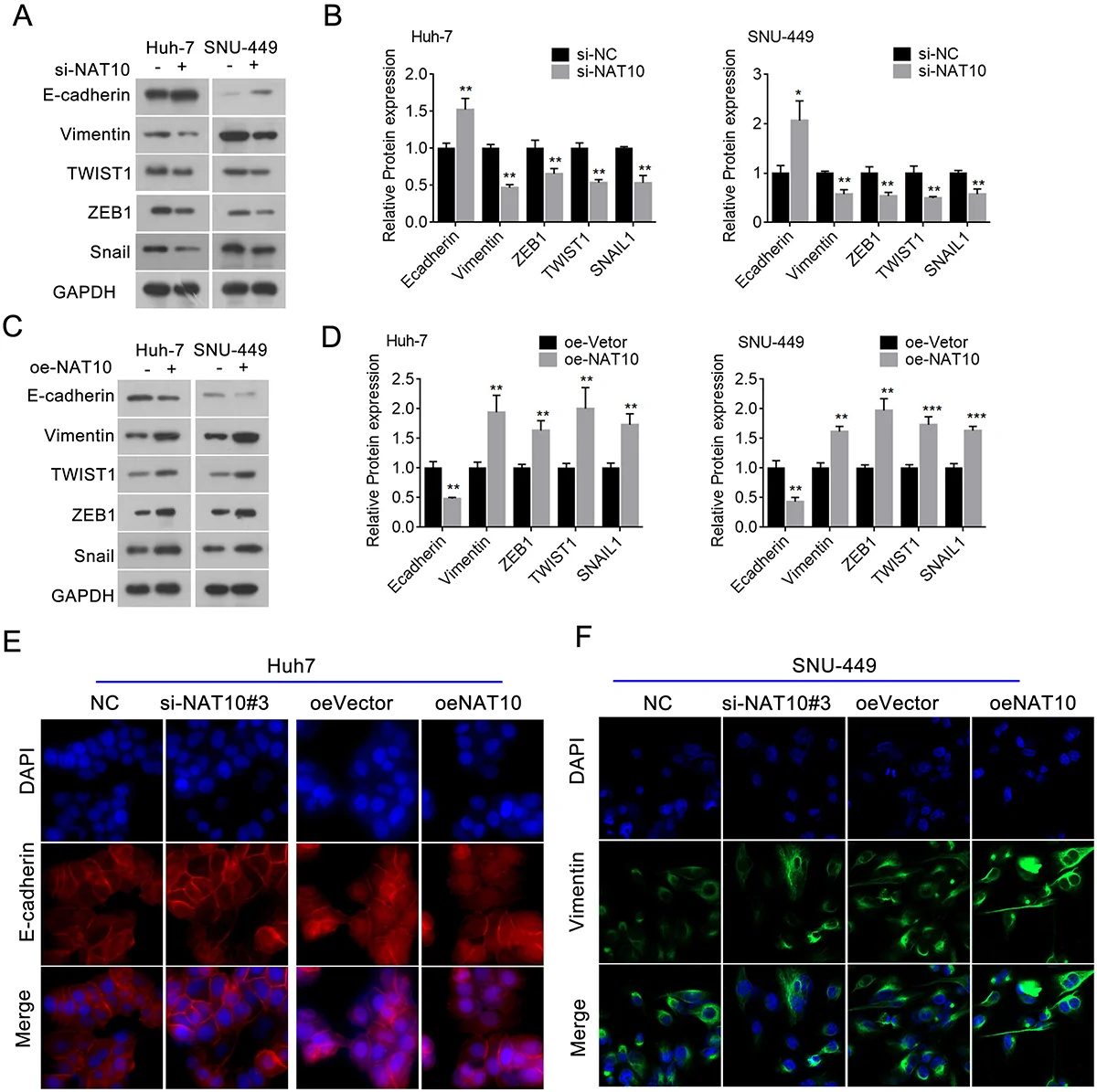
Effect of NAT10 on EMT in HCC cells. (A-B) Expression levels of EMT-related markers (Vimentin, Snail, TWIST, ZEB1, and E-cadherin) in NAT10-depleted cells (si-NAT10) compared to those in the negative control (NC). (C-D) Expression levels of EMT markers in NAT10-overexpression cells compared to those in the vector control. (E) Immunofluorescence analysis showing E-cadherin expression in NAT10-depleted HUH7 cells. (F) Immunofluorescence analysis showing Vimentin expression in NAT10-overexpression SNU-449 cells.

EMT, epithelial – mesenchymal transition; HCC, hepatocellular carcinoma; NAT10,*N*-acetyltransferase 10.

We next examined whether NAT10 influences EMT in HCC cells. Western blot analysis of EMT-related markers showed that NAT10 knockdown significantly increased the epithelial marker E-cadherin and concomitantly decreased the mesenchymal markers Vimentin, Snail, ZEB1, and Twist compared with the NC group (Figure 3(A–B)). In contrast, NAT10 overexpression suppressed E-cadherin expression and upregulated Vimentin, Snail, ZEB1, and Twist (Figure 3(C–D)). These findings were further corroborated by immunofluorescence staining. NAT10-depleted cells exhibited enhanced E-cadherin expression (Figure 3(E)), whereas NAT10-overexpressing cells displayed increased Vimentin expression (Figure 3(F)). These results demonstrate that NAT10 promotes EMT in HCC cells, favoring a mesenchymal phenotype that is closely associated with enhanced migratory and invasive capacities.

### NAT10 facilitates the growth and metastasis of HCC cells in vivo

**Figure 4.**
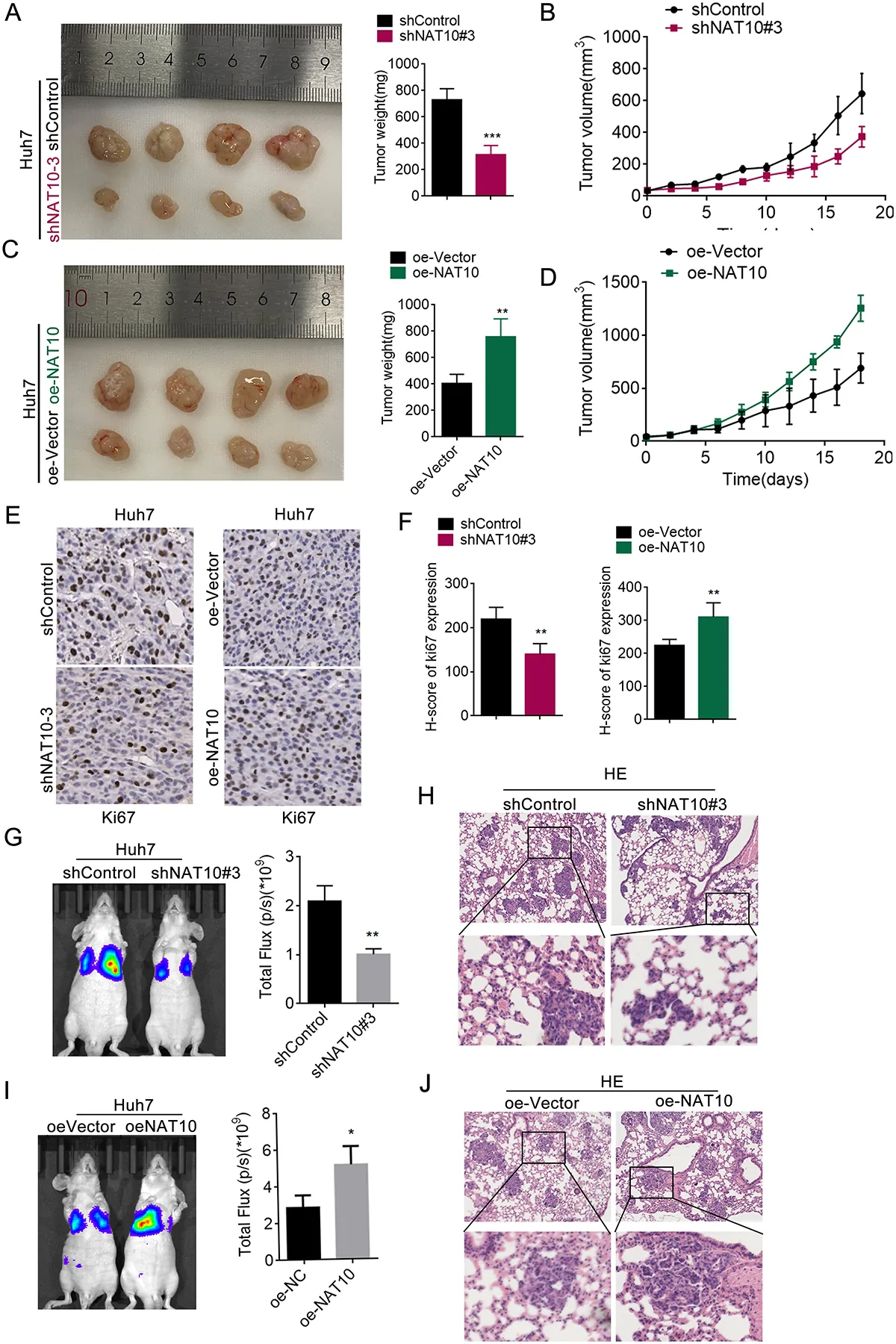
Effect of NAT10 on xenograft tumor growth and metastasis. (A, B) Tumor growth curves, final tumor volumes, and tumor weights in nude mice subcutaneously injected with Huh7-shNAT10 or control cells over an 18-day period. (C – D) Comparison of tumor volumes and weights between the NAT10-overexpression group and the vector group. (E – F) Immunohistochemistry analysis of Ki-67 expression in NAT10 shRNA and NAT10-overexpression groups. (G – I) Bioluminescent imaging of mice injected with luciferase-expressing HUH7 cells, including HUH7-shNAT10, HUH7-NAT10, HUH7-shcontrol-GFP, and HUH7-vector-GFP, to establish a lung metastasis model via the tail vein. Quantitative analysis of bioluminescent signals from different treatment groups. (H, J) Representative images of H&E-stained lung sections from each treatment group.

Next, we explored the influence of NAT10 on the proliferation of xenograft tumors in Huh7 cells. Initially, nude mice were randomly allocated into groups and received subcutaneous injections of Huh-7-NAT10, Huh7-shNAT10, or their respective control cells. Over the next 18 days, any alterations in tumor size, volume, and growth rate were meticulously monitored. As depicted in Figure 4(A), tumors arising from the NAT10 shRNA cohort were markedly smaller in comparison to that in the control group. Their weights were also considerably lower than those observed in the control group. The statistical analysis presented in the histogram further illustrated that tumor volumes in the NAT10 shRNA group were significantly reduced (Figure 4(B)). Conversely, the tumor dimensions, volumes, and weights in the NAT10-overexpression group were substantially elevated compared to those in the vector group (Figure 4(C-D)). To further substantiate the impact of NAT10 on the tumorigenic potential of HCC cells *in vivo*, immunohistochemical analysis was performed to assess Ki-67 expression levels. The findings indicated that Ki-67 expression in the NAT10 shRNA group was significantly diminished relative to that in the control group, whereas it was significantly augmented in the NAT10-overexpression group compared to that in the vector group (Figure 4(E-F)).

H&E, hematoxylin and eosin; NAT10,*N*-acetyltransferase 10.

To verify the function of NAT10 *in vivo*, we injected luciferase-expressing Huh7-shNAT10, Huh7-NAT10, Huh7-shcontrol, and Huh7-vector cells into the tail vein of mice to establish a lung metastasis model. The progression of lung metastasis was dynamically monitored weekly using an IVIS Lumina III imaging system. Bioluminescence imaging showed that NAT10 knockdown inhibited HCC lung metastases. Conversely, mice with NAT10 overexpression had a significantly higher luciferase activity than that in controls (Figures 4(G,I)). At the experimental endpoint, lung tissues were harvested and subjected to H&E staining. Fewer micrometastases were microscopically detected in the lungs of the NAT10-knockdown mice, while the NAT10-overexpression mice demonstrated significantly higher metastases (Figures 4(H,J)). These data prove that NAT10 may promote the growth and metastasis of HCC cells *in vivo*.

### NAT10 exerts its effect by α-tubulin acetylation

**Figure 5.**
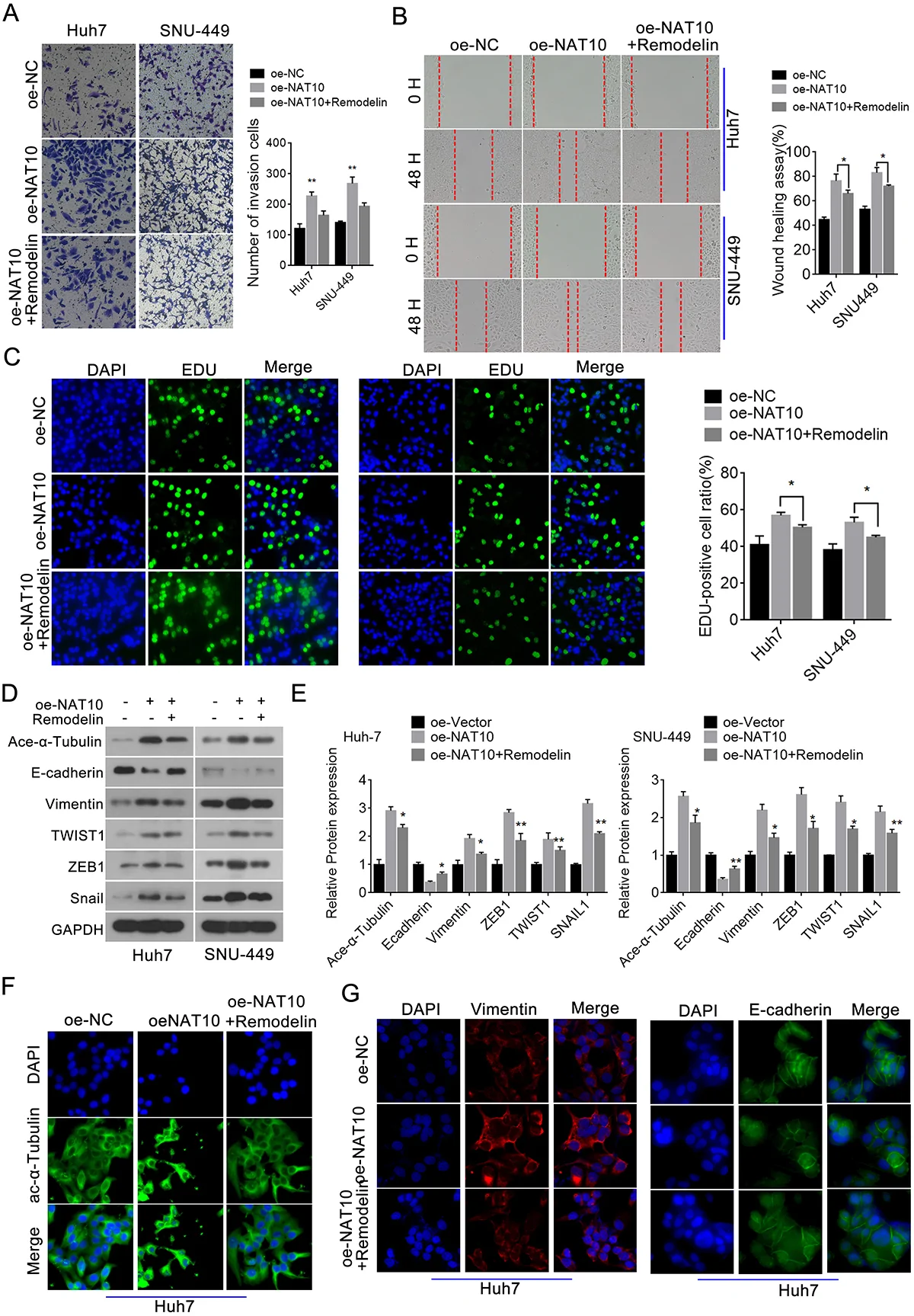
NAT10 impacts HCC cell behavior through α-tubulin acetylation. (A – C) Transwell invasion, wound healing migration, and EdU proliferation assays demonstrating the effects of NAT10 overexpression in HCC cells in the presence or absence of Remodelin. (D-E) Western blot analysis of acetylated α-tubulin and EMT levels in NAT10-overexpression HCC cells, with or without Remodelin. Immunofluorescence analysis of (F) acetylated tubulin, (G) Vimentin and E-cadherin in NAT10-overexpression HUH7 cells, with or without Remodelin.

Since NAT10 can influence tubulin acetylation, we aimed to determine whether it also affects cell proliferation, invasion, and migration of cancer cells and EMT through this mechanism. Remodelin, a pharmacological inhibitor of NAT10, was used to interrogate this mechanism. Based on dose – response analysis (Figure S3), 1 μM Remodelin was selected as the minimal effective concentration for subsequent experiments. HCC cells were treated with Remodelin and then transfected with the NAT10 overexpression plasmid to assess whether inhibition of NAT10 could counteract its oncogenic effects. Transwell and wound healing assays showed that the NAT10-overexpression group demonstrated a marked increase in both invasion and migration of HCC cells compared to that in the NC group, as evidenced by a higher number of cells migrating through the transwell membrane and a smaller wound area in the wound healing assay (Figure 5(A,B)). However, treatment with Remodelin significantly diminished these effects, with fewer cells migrating and a larger wound area in the NAT10 overexpression + Remodelin group compared to that in the NAT10-overexpression group (Figure 5(A,B)). Consistently, EdU assays showed that NAT10 overexpression significantly increased cell proliferation, whereas Remodelin substantially suppressed this proliferative advantage (Figure 5(C)). At the molecular level, Remodelin abrogated the NAT10-induced increase in acetylated α-tubulin and reversed EMT-associated changes, restoring E-cadherin expression while reducing Vimentin, Snail, ZEB1, and Twist levels (Figure 5(D,E)). Immunofluorescence analysis further confirmed these findings. In Huh7 cells, NAT10 overexpression elevated acetylated α-tubulin and Vimentin expression while suppressing E-cadherin; these alterations were partially reversed by Remodelin treatment (Figure 5(F,G)). These data indicate that NAT10 promotes proliferation, migration, invasion, and EMT in HCC cells primarily through enhancing α-tubulin acetylation.

EMT, epithelial – mesenchymal transition; NAT10,*N*-acetyltransferase 10.

## Discussion

This study shows that NAT10 promotes proliferation, invasion, and EMT in HCC cells through α-tubulin acetylation. Our findings indicate that NAT10 overexpression enhances α-tubulin acetylation and facilitates cell invasion, migration, and EMT characteristics, while its knockdown produces the opposite effects. Additionally, we demonstrated that NAT10 expression can influence tumor growth and metastasis *in vivo*, highlighting its critical role in HCC progression. Importantly, we found that Remodelin can reverse the effects of NAT10 overexpression, which further supports the idea that NAT10 acts through the acetylation of α-tubulin. This research not only underscores the significance of NAT10 in tumor biology, but also provides new avenues for clinical investigation, suggesting that NAT10 may serve as a novel therapeutic target to improve treatment outcomes in HCC.

EMT is a critical process in tumor progression and metastasis that has garnered significant attention in recent years. During EMT, epithelial cells acquire mesenchymal characteristics, enhancing their migratory and invasive capabilities. This phenomenon has been observed in various cancers, particularly in aggressive malignancies such as breast cancer [22] and colorectal cancer [23]. Our findings indicate that NAT10 overexpression can promote EMT, corroborating previous studies and underscoring the importance of EMT in the progression of HCC [13,24]. EMT is induced by different transcription factors commonly referred to as EMT transcription factors. Among these regulators, Snail plays a pivotal role in the EMT process. Snail is recognized as the most critical transcriptional repressor of E-cadherin, because it can directly bind to the E-box sequences in the promoter region of E-cadherin and inhibit its expression [25]. Our research demonstrates that when NAT10 expression is decreased, Snail, TWIST, and ZEB1 expression also diminishes; conversely, when its expression is elevated, Snail, TWIST, and ZEB1 expression levels increase correspondingly. These results support the hypothesis that NAT10 is a key regulatory factor in the EMT process within HCC cells, highlighting its potential role in promoting tumor invasiveness and progression.

A noteworthy finding of our study is the identification of α-tubulin acetylation as a mechanism through which NAT10 exerts its effects. Tubulin acetylation is a post-translational modification that can regulate microtubule structure and maintain microtubule integrity [26]. α-Tubulin acetylation has been implicated in microtubule stability, influencing cell motility and shape [27]. However, the role of microtubule stability in tumors is dualistic; it can inhibit tumor progression as well as promote malignant behavior. Some researchers argue that microtubule stability plays an anti-cancer role. For instance, the loss of Tektin4 promotes metastasis in triple-negative breast cancer by decreasing microtubule stability through HDAC6-mediated tubulin deacetylation [28]. Additionally, HTRA1 may facilitate the interaction between HDAC6 and α-tubulin, enhancing cell migration by decreasing α-tubulin acetylation in human glioblastoma cells [29]. RASSF1A, a tumor suppressor protein, increases α-tubulin acetylation by inhibiting HDAC6 activity, thereby stabilizing microtubules and suppressing the migration of lung cancer cells. This result suggests that high acetylation levels can have anti-cancer effects in this context [30]. Furthermore, panobinostat, an HDAC inhibitor, inhibits the growth of thyroid cancer cells by stabilizing acetylated microtubules [31]. In contrast, others suggest that microtubule stability may promote cancer progression. For example, in breast cancer, elevated levels of α-tubulin acetylation enhance the formation of microtentacles, which significantly increase tumor cell adhesion, invasion, and distant metastasis [32]. Similarly, in bone metastatic breast cancer cells, Runx2 enhances microtubule stability through the induction of α-tubulin acetylation, which promotes autophagy and tumor survival in the bone microenvironment [33]. In HCC, high levels of acetylation are associated with poor patient prognosis and reduced efficacy of eribulin [34]. Inhibiting acetylation (e.g., through ATAT1 knockdown or HDAC6 overexpression) can significantly enhance chemotherapy sensitivity. Our study results demonstrate that NAT10 overexpression enhances α-tubulin acetylation, which in turn promotes proliferation, migration, and EMT in HCC cells, while its knockdown has the opposite effect. Notably, Remodelin can partially reverse the effects of NAT10 overexpression.

From our perspective, these findings position NAT10 as a critical mediator linking cytoskeletal remodeling to liver cancer progression. Our results, together with previous evidence, indicate that the subcellular localization of NAT10 is as functionally relevant as its expression level. Specifically, redistribution of NAT10 from the nucleolus to the cytoplasm and plasma membrane has been associated with enhanced tumor cell migration and increased α-tubulin acetylation [16]. This observation suggests that evaluating NAT10 subcellular distribution, rather than relying solely on total expression levels, may improve prognostic stratification and inform therapeutic decision-making. Emerging data further indicate that inhibition of NAT10 can potentiate anti-tumor immunity by activating type I interferon signaling pathways [35]. These findings broaden the therapeutic implications of targeting NAT10, extending beyond the suppression of cancer cell motility to the potential enhancement of immunotherapeutic responsiveness. Such a dual mechanism is particularly relevant in the current treatment landscape of HCC, which has shifted from single-agent multikinase inhibitors toward immune checkpoint inhibitor – based combination strategies [36]. The growing integration of locoregional therapies with systemic immunotherapy and anti-angiogenic regimens [37] further underscores the importance of positioning NAT10 within combination-oriented therapeutic frameworks. In addition, as HCC is increasingly linked to metabolic dysfunction – associated steatotic liver disease and chronic inflammatory states [38,39], future translational studies should incorporate host metabolic context into patient stratification strategies. Given that treatment outcomes are also influenced by nutritional and inflammatory status [40], integrating real-world measures of patient fitness will be essential when advancing NAT10-targeted approaches toward clinical application.

Although our study provides mechanistic evidence supporting a role for NAT10 in HCC progression, several limitations should be considered. First, the use of immunodeficient nude mice precluded evaluation of tumor – immune microenvironment interactions. Future studies employing immunocompetent models will be necessary to determine whether NAT10 inhibition can synergize with immunotherapeutic strategies. Second, only two HCC cell lines, Huh-7 and SNU-449, were used for functional analyses. Although these lines were deliberately selected to represent divergent baseline NAT10 expression levels, they may not fully reflect the molecular heterogeneity of HCC. Validation in additional cell lines and patient-derived models would further strengthen the robustness and translational relevance of our findings. Third, the relatively small clinical sample size (*n* = 9) limits statistical power; larger, well-characterized cohorts are required to confirm the prognostic value of NAT10 and α-tubulin acetylation. Finally, while our data functionally support a role for NAT10 in regulating α-tubulin acetylation, direct biophysical evidence of their interaction, such as co-crystallization studies or proximity ligation assays, remains to be established and warrants further investigation.

## Conclusions

This study reveals that NAT10 promotes proliferation, invasion, and EMT in HCC cells through α-tubulin acetylation. Hence, NAT10 can be considered a potential therapeutic target. Future research should focus on gaining a more comprehensive understanding of the role of NAT10 in tumor biology and laying the groundwork for developing targeted therapeutic strategies against NAT10.

## Supplementary Material

supplement file.docx

## Data sharing statement

All data supporting the conclusions of this study are included within the article.
