# Supplementary material for: NAT10 promotes proliferation, invasion, and epithelial–mesenchymal transition in hepatocellular carcinoma through α-tubulin acetylation
Source: Cell Adh Migr. 2026 Aug 24;20(1):2697609. doi: 10.1080/19336918.2026.2697609 (PMC13505444; doi:10.1080/19336918.2026.2697609)
Supplement: Supplementary file 1 [file KCAM_A_2697609_SM4160.docx]

**Figure S1. Expression and prognostic significance of NAT10 in HCC.** (A) Comparison of NAT10 mRNA expression levels between HCC tissues (n = 371) and normal liver tissues (n = 50) based on data from the UALCAN database. (B) Kaplan–Meier survival analysis showing the association between NAT10 expression and overall survival in patients with HCC. (C–D) Western blot analysis of NAT10 protein expression in six HCC cell lines (SNU-449, Huh-7, SNU-387, Hep3B, SNU-475, and SK-Hep-1). (E) RT-qPCR analysis of NAT10 mRNA expression in the same panel of HCC cell lines.

**Figure S2. NAT10 and α-tubulin acetylation expression in HCC tissues.** (A–B) Western blot analysis of NAT10 and acetylated α-tubulin protein levels in paired HCC tissues and adjacent normal tissues.

**Figure S3. Dose–response analysis of Remodelin in HCC cell lines.** (A) SNU-449 and (B) Huh-7 cells were treated with increasing concentrations of Remodelin (0–8 μM) for 24 h, and cell viability was subsequently assessed.
